# Insomnia, sleep duration and incident Parkinson's disease in the Finnish population cohort

**DOI:** 10.1093/braincomms/fcag108

**Published:** 2026-03-26

**Authors:** Sonja Sulkava, Katri Kantojärvi, Jari Haukka, Tiina Paunio

**Affiliations:** Department of Psychiatry and SleepWell Research Programme, Faculty of Medicine, University of Helsinki and Helsinki University Hospital, Helsinki 00260, Finland; Department of Welfare Epidemiology and Monitoring, Finnish Institute for Health and Welfare, Helsinki 00271, Finland; Department of Clinical Genetics, Helsinki University Hospital, Helsinki 00250, Finland; Department of Psychiatry and SleepWell Research Programme, Faculty of Medicine, University of Helsinki and Helsinki University Hospital, Helsinki 00260, Finland; Department of Welfare Epidemiology and Monitoring, Finnish Institute for Health and Welfare, Helsinki 00271, Finland; Department of Public Health, University of Helsinki, Helsinki 00290, Finland; Department of Psychiatry and SleepWell Research Programme, Faculty of Medicine, University of Helsinki and Helsinki University Hospital, Helsinki 00260, Finland; Department of Welfare Epidemiology and Monitoring, Finnish Institute for Health and Welfare, Helsinki 00271, Finland

**Keywords:** Parkinson’s disease, prospective study, sleep disturbance, competing risks, genetic risk score

## Abstract

Sleep problems are common in Parkinson's disease. Several lines of research have linked insufficient sleep to neurodegenerative processes, raising the possibility that sleep disturbances may serve as aetiological risk factors for neurodegenerative diseases. However, most cohort studies on sleep traits and Parkinson's disease have been too short to disentangle risk factors from prodromal symptoms. This study investigated the association of insomnia and sleep duration with incident Parkinson's disease in a Finnish cohort with long follow-up and modelling for the competing risk of death. The study included 73 281 Parkinson's disease-free participants with a mean age of 45.7 (12.3) years from the National FINRISK Study surveys conducted every 5 years from 1972 to 2012. Insomnia (never, sometimes and often) and night-time sleep duration (<7 h, 7–8 h, >8 h) were self-reported at baseline and linked with health register data on incident Parkinson's disease. We applied both a cause-specific hazard model (Poisson) and a subdistribution hazard model (Fine–Gray) accounting for the competing risk of death. Additionally, we examined associations between polygenic risk scores for the sleep traits and Parkinson's disease. During 1 806 843 person-years of follow-up (mean 24.6 ± 12.4 years), 2679 participants developed Parkinson's disease. The cause-specific hazard model showed an association of insomnia reported as sometimes (IRR 1.14, 95% CI 1.05–1.24) and often (IRR 1.54, 95% CI 1.35–1.75) with Parkinson's disease, and a subdistribution hazard model showed an association of insomnia often (IRR 1.26, 95% CI 1.11–1.43). In sensitivity analyses excluding individuals with <20 years of follow-up, the association for insomnia symptoms often remained (IRR 1.37, 95% CI 1.13–1.66). Neither self-reported short nor long sleep duration was associated with Parkinson's disease. Accordingly, polygenic risk score for insomnia (IRR 1.13, 95% CI 1.05–1.23) but not for short or long sleep was associated with incident Parkinson's disease. In this long-term cohort study, both self-reported insomnia and genetic liability to insomnia were associated with an increased risk of incident Parkinson's disease, suggesting insomnia is a potential risk factor rather than solely a prodromal symptom. However, the attenuated association of insomnia with the cumulative incidence of Parkinson's disease in the subdistribution hazard model accounting for the competing risk of death suggests a modest real-life effect.

## Teaser

Sulkava *et al.* report association of insomnia symptoms and their genetic liability with incident Parkinson's disease in a Finnish population cohort with a mean follow-up time of 25 years. The results suggest that insomnia, but not sleep duration, may be a risk factor for Parkinson's disease and not only a prodromal symptom.

## Graphical Abstract

**Graphical Abstract:**
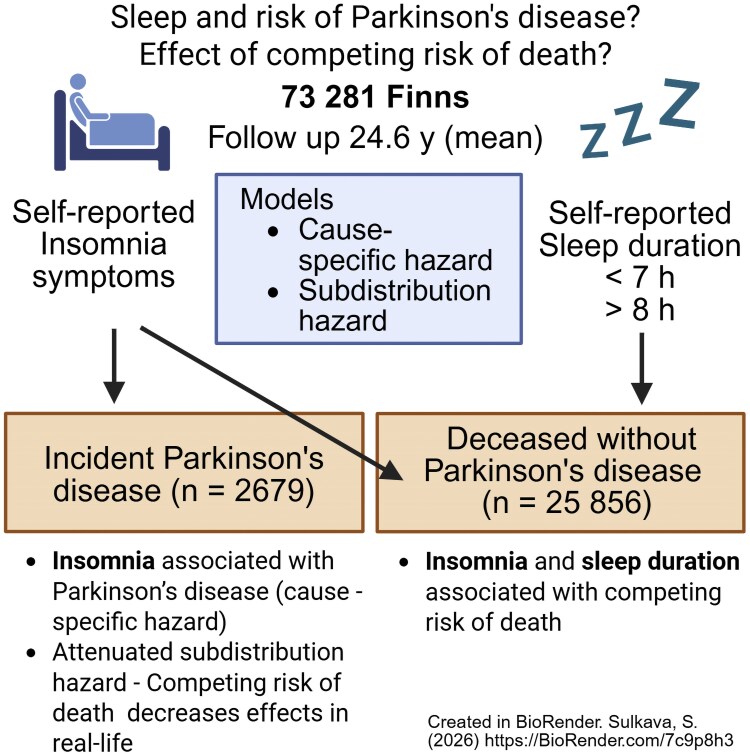

## Introduction

Parkinson's disease is one of the fastest-growing neurological conditions worldwide.^1^ Since there is no disease-modifying medication, prevention through modification of the risk factors is important. However, compared to another neurodegenerative disease, Alzheimer's disease,^2^ there is less research and evidence on modifiable risk factors.

Sleep disorders are common in Parkinson's disease; about 70% of individuals with Parkinson's disease suffer from insomnia, excessive daytime sleepiness, or REM sleep behaviour disorder (RBD) 5 years after diagnosis, with insomnia being the most frequent complaint.^3^ However, even before motor parkinsonism leads to clinical diagnosis, Parkinson's disease-related neuropathology can cause non-motor prodromal symptoms.^4^ Among the sleep disorders, RBD is an established prodromal symptom of Parkinson's disease, occurring several years to a few decades before diagnosis.^5^ Insomnia and worsening sleep quality have also been reported to precede Parkinson's disease diagnosis for several years.^6,7^

In addition to being potential prodromal symptoms, it has been suggested that sleep disturbances may promote the neurodegenerative process of Parkinson's disease through mechanisms such as glymphatic system dysfunction and increased neuroinflammation, and thus may serve as aetiological risk factors.^8^ However, even cohort studies with the longest follow-up times have likely been too short to separate prodromal symptoms from true risk factors, given that the neuropathological process of Parkinson's disease may begin decades before clinical diagnosis.^7,9-12^

In age-related diseases like Parkinson's disease, death from non-Parkinson's disease causes can serve as a competing risk as individuals may die before reaching the age at which Parkinson's disease is typically diagnosed. When studying mortality-associated explanatory variables, such as sleep traits, it is important to consider the competing risk of death to fully understand their relationship to disease risk and the potential for prevention through risk factor modification.^13^ This aspect has so far been overlooked in the epidemiological studies of Parkinson's disease.

In this study, we aimed to clarify whether insomnia symptoms or short or long night-time sleep are risk factors for Parkinson's disease. We used a large Finnish population cohort with a mean follow-up of 25 years and conducted sensitivity analyses limited to follow-up times longer than 20 years. In addition, we examined the association of sleep traits with the real cumulative incidence of Parkinson's disease in the presence of competing risk of death.

## Materials and methods

### Study population

The National FINRISK Study is a Finnish risk factor and health behaviour study consisting of independent cross-sectional surveys collected every 5 years from 1972 until 2012. Each survey consists of a random sample of inhabitants from selected regions of Finland aged 25 to 65 or 75 years. This prospective study combined FINRISK surveys from the years 1972, 1977, 1982, 1987, 1992, 1997, 2002, 2007 and 2012, with data on incident Parkinson's disease from the Finnish health registers. The study cohorts are described in detail in previous publications.^14,15^

The ethical committee of the Finnish Institute of Health and Welfare and/or the Coordinating Ethical Committee of Helsinki and Uusimaa Hospital District gave permissions for the FINRISK surveys 1992–2012. An ethic committee assessment was not required in Finland before the FINRISK survey 1992. However, declaration of Helsinki was followed and confidentiality, anonymity, and data protection have been assured according to Finnish legislation, and informed written consents were obtained from 1997 onward.

The FINRISK surveys 1972–2012 had altogether 99 259 invitees.^14^ Of them, 75 080 (75.6%) participated in the study and gave permission for the linkage to the health register data. In this study, first, 169 were excluded due to prevalent Parkinson's disease, and then 1630 due to missing information on the educational class, and 1563 individuals with missing information on insomnia, resulting in 71 718 participants for the analysis of insomnia. The sleep duration was questioned in the FINRISK 1972–1977 and 2007–2012 surveys, including 36 109 individuals. After the exclusion of 102 individuals due to prevalent Parkinson's disease, 610 individuals with missing information on educational class, and 465 individuals with missing information on sleep duration, 34 932 individuals were eligible for the analysis.

### Follow-up and ascertainment of Parkinson's disease

Follow-up continued from the baseline FINRISK survey until the diagnosis of Parkinson's disease, death (competing risk), or the end of the year 2019 (censoring), whichever occurred first. The potential follow-up time varied according to the FINRISK survey from 47 years (FINRISK 1972) to 7 years (FINRISK 2012).

Diagnosis of Parkinson's disease was received from the combined Finnish health registers: the Hospital Discharge Register and the Causes of Death Register (ICD-10: G20, ICD-9: 3320A, ICD-8: 34200), the Drug Reimbursement Register (code 110 Parkinson's disease and comparable movement disorders and diagnosis of Parkinson's disease, G20/3320A, as the reason for reimbursement). To be eligible for reimbursement in Finland, a medical certificate from a specialist doctor or from specialized health care with an adequate description of the case is needed. Individuals with a diagnosis of Parkinson's disease before the study entry (*n* = 169) were considered prevalent and were excluded from analyses. The number of incident Parkinson's disease cases was 2679.

### Assessment of sleep

Experience of insomnia symptoms was assessed in all FR cohorts with the question: ‘Think of the past month. Please mark the alternative which best describes how often the asked thing or symptom has been on your mind. Do you suffer from insomnia?’ The answer options were 1 = often, 2 = sometimes, and 3 = not at all. For the analyses, the scale was reversed. The night sleep duration was questioned in the FINRISK surveys 1972–1977 and 2007–2012 with the question: ‘How many hours on average do you sleep in one night?’ In 1972–2007, the answers were in hours and 2012, hours and minutes. The sleep length was categorized as short sleep (< 7 h), long sleep (>8 h) and reference (7–8 h). The category of very long night-time sleep in the secondary analyses was defined as >9 h.

### Assessment of potential confounders

Cardiovascular risk factors included systolic blood pressure (average of 1 to 3 measurements depending on the survey), body mass index (BMI), total cholesterol, self-reported smoking (non-smoker, current smoker or ex-smoker), physical activity as combined occupational, leisure, and commuting activity (low, moderate, or high as described previously^16,17^), and self-reported diabetes.

Depressive mood and nervousness were assessed as part of the same question pattern with insomnia question ‘Think of the past month. Please mark the alternative which best describes how often the asked thing or symptom has been on your mind. Do you feel depressed? or Do you feel tense and nervous?’, respectively. The answer options were 1 = often, 2 = sometimes, and 3 = not at all. For the analyses, the scale was reversed.

Sleep medication was assessed in the FINRISK surveys 1992–2007 with the question: ‘When was the last time you used the following medication: Sleeping pills’. The answer options were ‘1. During the past week, 2. 1–4 weeks ago, 3. 1–12 months ago, 4. Over a year ago, 5. Never. In 2012, the question also included benzodiazepine receptor agonists (Z-drugs). Responses were dichotomized to separate 1. Never use (5), and 2. Using now or in the past (1–4).

### Polygenic risk scores

Genome-wide genotyping and imputation methods for the FINRISK surveys are described in.^18^ The insomnia polygenic risk scores (PRS) were derived from UK Biobank GWAS data (109 548 cases, 277 440 controls), where insomnia was identified through a validated survey question serving as a reliable proxy for insomnia disorder.^19^ The short sleep PRS, long sleep PRS and sleep duration PRS were constructed from UK Biobank GWAS data (*N* = 446 118), based on self-reported habitual sleep duration (<7 h: short sleep and ≥9 h: long sleep), supplemented with accelerometer data from 85 499 participants.^20^ Each PRS included approximately 1 million variants, standardized to a mean of 0 and SD of 1 using R 3.6.0 software.

The PRSs were produced with the PRS-CS method that infers posterior SNP effect sizes under continuous shrinkage priors using the European 1000 Genomes Project as the LD reference panel.^21^ Top 10 genetic population components based on the GWAS data were provided with the PLINK2 program (www.cog-genomics.org/plink/2.0/0).^22^

### Statistical analyses

Incident Parkinson's disease and death without Parkinson's disease (competing risk of death) were considered as competing risks. To study the association of sleep traits with incident Parkinson's disease in our primary analyses, we used in parallel two models accounting for competing risk: the Poisson regression model as a cause-specific hazard model and the Fine–Gray model as a subdistribution hazard model, similarly to the previous publication.^15^ The cause-specific hazard model is considered to emphasize aetiological risk factors as the subdistribution hazard model estimates the effect on cumulative incidence.^13,23^ Poisson regression model was favoured over Cox regression model as it better fits our study setting with several relevant time-scales that should be modelled (biological age, study year and follow-up time).

Follow-up time and age at the end of follow-up were split into fixed time bands and used as covariates in the cause-specific hazard model (Poisson). The break points for follow-up time were 13, 23 and 33 years, and the break points for age were 50, 60, 65, 70, 75, 80 and 85. In addition, covariates of sex, educational class and survey year were used in the primary analyses.

Sensitivity analyses to reduce reverse causation excluded individuals with less than 20 years of follow-up. They were performed for the traits that showed association with Parkinson's disease in hazard models.

We performed several secondary analyses using cause-specific hazard models. The effect of additional covariates, cardiovascular risk factors, depressive mood, nervousness and sleep medication was studied. The primary analyses were also repeated by baseline age groups (<35, 35–50, 50–65, and >65 years). To test interactions with sex, we added an interaction term of sex × insomnia and sex × sleep duration classes to the Poisson model. To study the effect of the missing information, we repeated our main analyses and included the category of no response. *E*-values were calculated with the following equation. *E*-value = IRR + sqrt(IRR*(IRR − 1)), IRR = incidence rate ratio.^24^

In addition, as another independent sleep-related measure, an association of Parkinson's disease with polygenic risk scores of insomnia, sleep duration, short sleep and long sleep was studied using a cause-specific hazard model. The first three genetic population components from the GWAS data, follow-up time and age at the end of follow-up were used as covariates in the analyses.

95% confidence intervals (CI) and 2-sided testing were used throughout the study. R software version 4.0.0 (R Project for Statistical Computing), version 4.2.2 (other than hazard models), and RStudio version 2022.07.2 were used with packages Epi, cmprsk, dplyr and survival.

## Results

### Basic characteristics

Supplementary Table 1 summarizes baseline characteristics and incident outcomes by insomnia and sleep duration categories. Individuals reporting insomnia symptoms often were older, less educated and more often women in comparison to those who reported never suffering from insomnia. They tended to have more unfavourable cardiovascular risk profiles, and more often depressive mood, and nervousness. They were more often members of the newest FINRISK cohorts, and they had shorter follow-up times. As expected, they had higher insomnia and short sleep PRS scores and lower sleep duration PRS score. The profile of the short sleep group resembled that of individuals with insomnia, except that the number of men and women was equal. The long sleep group was characterized by female prominence and slightly lower educational class compared to the 7–8 h group. Some of the cardiovascular risk factors were more unfavourable compared to the 7–8 h group, but less than in the insomnia often or short sleep group. The long sleep PRS and sleep duration PRS were higher and the short-sleep PRS lower compared to the 7–8 h group.

**Figure 1:**
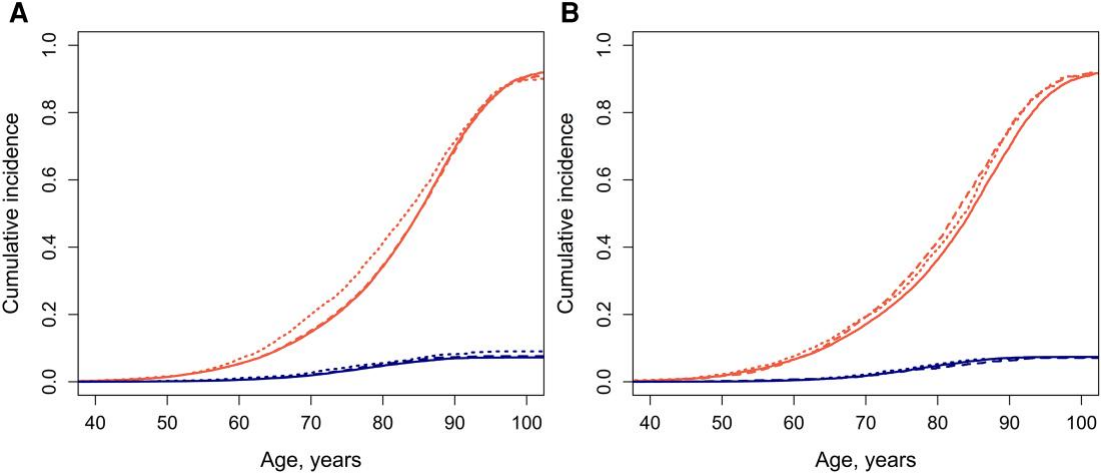
**Cumulative incidences of Parkinson's disease and competing risk of death (Parkinson's disease-free death).** Insomnia **(A)**. *n* = 71 718. Red: competing risk of death. Blue: Parkinson's disease. Solid line, insomnia never; dashed line, insomnia sometimes; dotted line, insomnia often. Sleep duration categories **(B)**. *n* = 34 932. Red: competing risk of death. Blue: Parkinson's disease. Solid line, normal sleep; dashed line, short sleep; dotted line, long sleep.

There were 2679 incident Parkinson's disease cases over 1 806 843 person-years of follow-up. The mean follow-up time was 24.6 (12.4) years. The overall incident rate per 100 000 person-years was 161, being 239 for those with insomnia often, 130 for those with insomnia never. When studying sleep duration restricted to FINRISK cohorts 1972–1978 and 2007–2012 the incidence rate was 161 for short sleepers, and 144 for long sleepers. Figure 1 shows the cumulative incidences of Parkinson's disease and the competing risk of death (death without Parkinson's disease) by insomnia and sleep duration categories.

### Association of sleep traits with Parkinson's disease: hazard models

In the primary analyses, we modelled in parallel cause-specific hazard model and subdistribution hazard model for both Parkinson's disease and competing risk of death (Table 1). Cause-specific hazard model showed an association of insomnia sometimes (incidence rate ratio [IRR] 1.14, 95% CI 1.05–1.24) and often (IRR 1.54, 95% CI 1.35–1.75) with Parkinson's disease in a dose-response manner, but no association of short (IRR 1.00, 95% CI 0.84–1.18) or long sleep duration (IRR 1.01, 95% CI 0.86–1.20). In the subdistribution hazard model, only insomnia often showed an association with Parkinson's disease (IRR 1.26, 95% CI 1.11–1.43). Both insomnia symptoms and sleep duration classes showed associations with the competing risk of death.

**Table 1:** Associations of sleep characteristics with Parkinson's disease

	All	Parkinson's disease	Parkinson's disease		Competing risk of death	
	No.	No.	Cause-specific hazard model (Poisson)^a^	Subdistribution hazard model (Fine–Gray)^b^	Cause-specific hazard model (Poisson)^a^	Subdistribution hazard model (Fine–Gray)^b^
Trait			IRR (95% CI)	HR (95% CI)	IRR (95% CI)	HR (95% CI)
**Insomnia** Never	41 314	1416	1	1	1	1
Sometimes	24 749	933	1.14 (1.05–1.24)	1.06 (0.98–1.15)	1.12 (1.10–1.14)	1.13 (1.10–1.16)
Often	5655	280	1.54 (1.35–1.75)	1.26 (1.11–1.43)	1.39 (1.35–1.43)	1.40 (1.34–1.46)
**Night sleep duration** <7.0 h	4938	165	1.00 (0.84–1.18)	0.87 (0.73–1.02)^c^	1.25 (1.21–1.29)	1.20 (1.14–1.25)^c^
7–8 h	25 589	1045	1	1	1	1
>8.0 h	4405	159	1.01 (0.86–1.20)	0.92 (0.78–1.09)^c^	1.19 (1.15–1.23)	1.16 (1.10–1.22)^c^

Cause-specific hazard model (Poisson) and subdistribution hazard model (Fine–Gray).

CI, confidence interval; HR, hazard ratio; IRR, incidence rate ratio.

^a^Model adjusted for sex, educational class, survey year, follow-up time (10-year intervals), and age at the end of follow-up (5-year intervals).

^b^Model adjusted for sex, educational class, and survey year.

^c^Covariate of survey year could not be included for numerical reasons.

**Figure 2:**
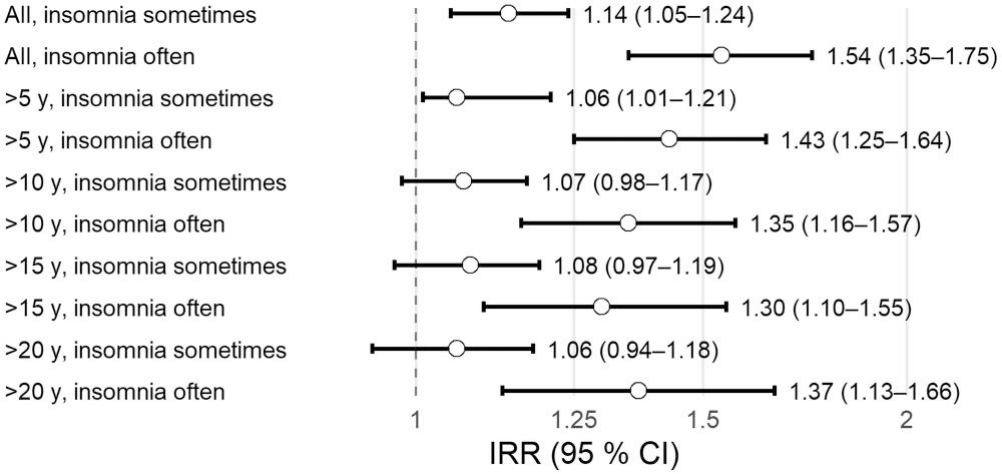
**Forest plot showing the primary and sensitivity analyses of the association between insomnia and incident Parkinson's disease using cause-specific hazard model (Poisson).** y, years; *z*, *z*-statistic; *P*, *P*-value. All *n* (cases) = 71 718 (2629), *z* = 3.12, *P* = 0.002, *z* = 6.46, *P* = 1.07E-10; >5 y *n* (cases) = 69 858 (2470), *z* = 2.27, *P* = 0.02, *z* = 4.53, *P* = 5.9E-6; >10 y *n* (cases) = 61 769 (2172), *z* = 1.43, *P* = 0.15, *z* = 3.95, *P* = 7.8E-5; >15 y *n* (cases) = 53 132 (1835), *z* = 1.43, *P* = 0.15, *z* = 3.08, *P* = 0.002; >20 y *n* (cases) = 42 395 (1467), *z* = 0.94, *P* = 0.35, *z* = 3.19, *P* = 0.001.

Sensitivity analyses for Parkinson's disease in the cause-specific hazard model, including individuals with at least 20 years of follow-up, showed association with insomnia often (IRR 1.37, 95% CI 1.13–1.66) but not with insomnia sometimes (IRR 1.06, 95% CI 0.94–1.18) (Fig. 2). Further sensitivity analyses showed similar effect sizes for insomnia often when using 10-, 15- and 20-year cut-off points (Fig. 2).

### Association of sleep PRSs with Parkinson's disease

As independent proxy measures for sleep, we studied insomnia and sleep duration PRSs and their association with Parkinson's disease (Table 2). Similarly to our main analyses, a measure for insomnia (insomnia PRS) but not sleep duration (sleep duration, short sleep and long sleep PRS) showed an association with Parkinson's disease in the cause-specific hazard model.

**Table 2:** Association of sleep polygenic risk scores with Parkinson's disease

Trait	IRR (95% CI)
Insomnia PRS	1.13 (1.05–1.23)
Sleep duration PRS	1.02 (0.95–1.11)
Short sleep PRS	1.00 (0.92–1.08)
Long Sleep PRS	1.05 (0.97–1.14)

Cause-specific hazard model (Poisson) with 27 109 individuals, 631 with Parkinson's disease.

CI, confidence interval; IRR, incidence rate ratio.

Model adjusted for sex, educational class, survey year, follow-up time (10-year intervals), age at the end of follow-up (5-year intervals), and population components C1–C3.

### Secondary analyses

Association of sleep length with Parkinson's disease was studied further by focusing on very long sleep (>9 h). The very long sleep showed an association with Parkinson's disease (IRR 1.44, 95% CI 1.04–1.99). The association did not remain in the sensitivity analysis restricted to individuals with over 20 years of follow-up (Supplementary Table 2).

Further secondary analyses showed that the association of insomnia symptoms often with Parkinson's disease remained in the cause-specific hazard model when adjusting for cardiovascular risk factors, depressive mood, self-reported sleep medication and nervousness (Table 3). However, IRR was lower (1.39, 95% CI 1.20–1.61) when adjusting for nervousness and higher when adjusting for sleep medication (1.77, 95% CI 1.35–2.25). Including non-respondents as a separate class did not affect the association of sleep traits with Parkinson's disease. Individuals with no response on sleep traits showed an association with the competing risk of death but not with Parkinson's disease (Supplementary Table 3). The effect of potential unmeasured confounding on the association of insomnia with Parkinson's disease was estimated with the *E*-value. For the insomnia sometimes the *E*-value was 1.54 and for the insomnia often 2.45, suggesting that an unmeasured confounder with a risk ratio higher than that would be needed to nullify the observed associations.

**Table 3:** Association of insomnia with Parkinson's disease adjusted for cardiovascular risk factors, depressive mood, nervousness and sleep medication

	Model adjusted for cardiovascular risk factors^a^			Model adjusted for depressive mood			Model adjusted for nervousness			Model adjusted for sleep medication		
Trait	No., all	No., PD	IRR (95% CI)	No., all	No., PD	IRR (95% CI)	No., all	No., PD	IRR (95% CI)	No., all	No., PD	IRR (95% CI)
**Insomnia** Never	36 652	1277	1	35 927	1350	1	35 959	1354	1	14 114	239	1
Sometimes	21 465	807	1.11 (1.01–1.21)	19 184	818	1.08 (0.99–1.19)	19 336	831	1.08 (0.99–1.18)	10 892	254	1.14 (0.95–1.38)
Often	4788	246	1.54 (1.34–1.77)	4462	249	1.47 (1.26–1.70)	4497	250	1.39 (1.20–1.61)	2420	113	1.74 (1.35–2.25)
**Night sleep time**												
<7.0 h	21 413	928	0.96 (0.80–1.15)	17 401	922	0.88 (0.73–1.07)	17 545	932	0.88 (0.72–1.06)	7632	100	1.32 (0.92–1.91)
7–8 h	3937	135	1	2711	117	1	2746	120	1	2062	42	1
>8.0 h	3615	139	1.00 (0.84–1.20)	2875	132	0.98 (0.82–1.18)	2897	133	0.98 (0.82–1.18)	1391	21	1.25 (0.78–2.02)

Cause-specific hazard model (Poisson).

CI, confidence interval; IRR, incidence rate ratio; PD, Parkinson's disease.

Model adjusted for sex, educational class, survey year, follow-up time (10-year intervals), and age at the end of follow-up (5-year intervals).

^a^Body mass index, total cholesterol, mean systolic blood pressure, smoking, physical activity, and diabetes.

The FINRISK study subjects represented different ages. Therefore, the association of insomnia with Parkinson's disease was studied further by stratifying the analyses based on the baseline age (Supplementary Table 4). In the insomnia often group, slightly higher IRRs were seen in the younger baseline age groups, so that IRR 1.66 (95% CI 1.01–2.71) for the individuals less than 35 years in the baseline was followed by IRRs 1.52 (95% CI 1.20–1.92) and 1.55 (1.29–1.86) for those between 35–50 and 50–65 years and IRR 1.44 (0.92–2.27) for individuals over 65 years. There was no interaction between sex and sleep traits in relation to Parkinson's disease (Supplementary Table 5).

## Discussion

In this study, we found a robust association of insomnia symptoms at baseline with subsequent Parkinson's disease. The association remained, slightly reduced, in the sensitivity analysis, which included participants with at least a 20-year follow-up period. Association over such a long time suggests that insomnia would not only be a prodromal symptom but also a risk factor for Parkinson's disease. This conclusion is also supported by the association of insomnia PRS with incident Parkinson's disease.

In contrast, neither short nor long sleep duration nor their PRS showed an association with Parkinson's disease. However, as with insomnia, both short and long sleep durations showed an association with the competing risk of death, in line with the U-shaped curve between sleep duration and mortality shown before in the National FINRISK study^25^ and elsewhere.^26^ In addition, the very long night-time sleep, >9 h, showed association with increased risk of Parkinson's disease, but that did not remain in the sensitivity analysis, possibly reflecting reverse causation with very long sleep as a marker of disease.

The cause-specific hazard model showed a stronger association between insomnia and Parkinson's disease than the sub-distribution hazard model (54% versus 26% increased risk for insomnia often). This is likely explained by the association of insomnia with the competing risk of death, which affects the real cumulative incidence, modelled in the sub-distribution hazard analysis, but not in the rate of the occurrence of Parkinson's disease in the individuals who are currently event-free, modelled in the cause-specific hazard analysis.^13^ Thus, despite being a risk factor for Parkinson's disease, the association of insomnia with the real cumulative incidence of Parkinson's disease is modest in the presence of the possibility of dying from other causes. Treating insomnia may not reduce the amount of Parkinson's disease in the population substantially because it may also reduce mortality due to other causes and increase the possibility of living long enough to get Parkinson's disease. However, when considering mortality and morbidity more widely, our study supports the importance of treating insomnia adequately.^27^

The association of insomnia or poor sleep quality with Parkinson's disease has been studied in a few cohorts with an average follow-up time of around 10 years^7,10-12,28^ and evaluated in a systematic review.^29^ The results have been conflicting, showing both increased and decreased risks of Parkinson's disease or non-significant results. These studies have not analysed separately individuals with at least 10 years of follow-up. Nigrostriatal degeneration of Parkinson's disease and symptoms of olfaction have been estimated to begin over 20 years before the clinical diagnosis,^9^ thus with shorter studies it is hard to separate prodromal symptoms of Parkinson's disease from risk factors.

The recent systematic review on epidemiological studies evaluating sleep features and subsequent neurodegenerative diseases showed an association of shorter and longer than normal sleep duration with Parkinson's disease.^29^ However, there is a noticeable trend that the studies with the longest follow-up show increased risk of Parkinson's disease in association with long sleep only.^10,30,31^ They all include individuals with 9 h night-time sleep in the category of long sleep as in our primary analysis. Similarly to studies on insomnia, studies on sleep duration have not separated individuals with over 10 years of follow-up.

Our results on the association of insomnia and insomnia PRS with Parkinson's disease are conflicting with previous Mendelian randomization studies, which show no connection between insomnia, or other sleep traits, and Parkinson's disease.^32-35^ PRS and Mendelian randomization approaches are quite similar in studying the effect of genetic exposure on outcome.^36^ However, there are several potential explanations for the discrepancy. The Mendelian randomization studies, which are considered having less confounding and reverse causation than observational studies, are, however, vulnerable to selection bias due to competing risks when studying late-onset disease.^37,38^ It is thus possible that the competing risk of death weakens the connection of insomnia and Parkinson's disease in the Mendelian randomization studies, while our cause-specific hazard analysis is not specifically vulnerable to that. In addition to the selection bias, the relatively low number of genetic markers used in the Mendelian randomization instrument (13–48 SNPs in the discussed studies), and a low explanatory power may make the studies underpowered to detect the association. On the other hand, PRS with many genetic markers in our study may be more vulnerable to horizontal pleiotropic effects, genetic markers affecting the outcome through other traits than the exposure of interest.^36^

Mechanisms explaining the association of insomnia but not sleep length with Parkinson's disease would include hyperarousal and reduced sleep quality associated with insomnia. Chronic insomnia is considered a state of persistent hyperarousal, including physiological hyperarousal with hyperactivity of the HPA axis during both day and night.^39,40^ Physiological stress may contribute to the development of Parkinson's disease through, for example, increased inflammation and oxidative stress, unfavourable metabolic changes, or changes in the gut microbiome.^41^ Insomnia affects sleep quality, also according to objective sleep measures. Greater sleep fragmentation, especially during REM sleep, has been reported,^42^ as well as increased transition probability from N2 to N1 sleep or wakefulness.^43^ Differences in sleep microstructure have also been reported, such as a greater amount of high-frequency EEG activity indicating cortical arousal.^44,45^ Some studies suggest that insomnia is associated with a reduced amount of slow-wave sleep, which could impair sleep-related recovery processes and thereby contribute to neurodegeneration. Poor sleep quality may impair the function of glymphatic clearance, potentially leading to the accumulation of abnormal proteins, including extracellular α-synuclein, a neuronal protein linked to Parkinson's disease and other synucleinopathies.^46^ Consistent with this, a study by Sohail *et al*. found that greater sleep fragmentation in older adults without Parkinson's disease was associated with a higher amount of Parkinson's disease pathology in the post-mortem brain.^47^

### Limitations

The association of insomnia with Parkinson's disease was not dependent on cardiological risk factors, depressive mood or sleep medication, while the strength of the association was reduced when controlling for nervousness, which can be directly linked to insomnia hyperarousal. However, we cannot rule out that part of the observed relation would be due to unmeasured confounding. For example, we could not study sleep apnoea or restless legs syndrome, which are candidate risk or prodromal factors for Parkinson's disease and are tightly linked to insomnia.^48,49^ The association of insomnia with Parkinson's disease is not likely to be confounded by RBD, as RBD patients do not typically suffer from insomnia.^50,51^  *E*-value for cause-specific hazard model of association of insomnia often with Parkinson's disease was 2.54. Thus, an effect size higher than that for the association of an unmeasured confounder with both insomnia and Parkinson's disease would be required to nullify the observed association.^24^ We consider this unlikely: In most studies on risk factors for Parkinson's disease lower risk ratios have been reported,^52^ and the most confirmed risk factors are either not likely to affect insomnia, like pesticides or would cause inverse association like tobacco or caffeine use, which are linked to a lower risk of Parkinson's disease but a higher risk of sleep problems.

An evident limitation of this study is the use of a single one-point question on insomnia and subjective sleep length instead of a validated sleep questionnaire and objective sleep measurement. It is, however, also noteworthy that with such a simple question on the subjective experience of insomnia without diagnostic evaluation, it was possible to identify individuals with over 50% increase in Parkinson's disease risk.

## Conclusion

Our results show a robust association of insomnia symptoms but not short or long sleep with Parkinson's disease using a large Finnish population cohort. Persistence of the association in the sensitivity analysis with over 20 years of follow-up and replication of the association with insomnia PRS suggest that insomnia would be an aetiological risk factor and not only a prodromal symptom for Parkinson's disease.

## Supplementary Material

fcag108_Supplementary_Data

## Data Availability

The data contain sensitive health information of included individuals, and are not publicly available, but National FINRISK study 1992–2012 data supporting conclusions of this study are available from the THL Biobank on application https://thl.fi/en/research-and-development/thl-biobank/for-researchers/application-process.
