# Supplementary material for: Insomnia, sleep duration and incident Parkinson's disease in the Finnish population cohort
Source: Brain Commun. 2026 Mar 26;8(2):fcag108. doi: 10.1093/braincomms/fcag108 (PMC13061589; doi:10.1093/braincomms/fcag108)
Supplement: fcag108_Supplementary_Data [file fcag108_supplementary_data.pdf]

Supplementary tables

Supplementary Table S1. Baseline descriptive statistics and outcomes by insomnia and sleep duration categories in the study sample[a].

		Insomnia[a]			Night Sleep duration[b]		
**Participant characteristics**	**Overall participants**	**never**	**sometimes**	**often**	**7-8 h**	**short, < 7 h**	**long, >8h**
**N, all**	73 281	41 314	24 749	5655	25 589	4938	4405
**Incident PD**							
No	70 602 (96.3)	39 898 (96.6)	23 816 (96.2)	5375 (95.0)	24 544 (95.9)	4773 (96.7)	4246 (96.4)
Yes	2679 (3.7)	1416 (3.4)	933 (3.8)	280 (5.0)	1045 (4.1)	165 (3.3)	159 (3.6)
**Deceased**							
No	45 975 (62.8)	26 887 (65.1)	15 475 (62.5)	2930 (51.8)	14 127 (55.2)	2612 (52.9)	2446 (55.5)
Yes	27 306 (37.2)	14 427 (34.9)	9274 (37.5)	2725 (48.2)	11 462 (44.8)	2326 (47.1)	1959 (44.5)
**Competing event (deceased without PD)**							
No	44 746 (63.4)	26 237 (65.8)	15 052 (63.2)	2791 (51.9)	13 738 (56.0)	2549 (53.4)	2370 (55.8)
Yes	25 856 (36.6)	13 661 (34.2)	8764 (36.8)	2584 (48.1)	10 806 (44.0)	2224(46.6)	1876(44.2)
**Baseline age, *y***	45.7 (12.3)	43.6 (12.1)	47.6 (12.2)	50.7 (11.5)	44.7 (12.1)	49.2 (12.2)	44.7 (13.3)
**Age at the end of follow-up, *y***	70.3 (13.1)	70.0 (13.0)	70.3 (13.3)	71.3 (12.9)	71.2 (14.1)	69.9 (14.1)	69.6 (14.7)
**Follow-up time, to censoring or to event, y**	24.6 (12.4)	26.4 (12.5)	22.7 (12.0)	20.6 (11.5)	26.6 (15.3)	20.7 (14.0)	25.0 (15.9)
**Educational class**							
low	21 299 (29.1)	11 708 (28.3)	7100 (28.7)	1838 (32.5)	6745 (26.4)	1576 (31.9)	9803 (30.0)
intermediate	25 177 (34.4)	14 209 (34.4)	8492 (34.3)	1932 (34.2)	9041 (35.3)	1790 (36.2)	1572 (34.8)
high	26 805 (36.6)	15 397 (37.3)	9157 (37.0)	1885 (33.3)	9803 (38.3)	1533 (31.8)	1507 (34.2)
**Sex**							
Female	37 894 (51.7)	20 276 (49.1)	13 537 (54.7)	3254 (57.5)	13 065 (51.1)	2470 (50.0)	2569 (58.3)
Male	35 387 (48.3)	21 038 (50.9)	11 212 (45.3)	2401 (42.5)	12 524 (48.9)	2468 (50.0)	1836 (41.7)
**Survey year**							
1972	11 528 (15.7)	7014 (17.0)	3430 (13.9)	851 (15.0)	8620 (33.7)	1276 (25.8)	1523 (34.6)
1977	12 024 (16.4)	7426 (18.0)	3557 (14.4)	906 (16.0)	9072 (35.5)	1496 (30.3)	1412 (32.1)
1982	9069 (12.4)	5554 (13.4)	2660 (10.7)	613 (10.8)	-	-	-
1987	6156 (8.4)	3500 (8.5)	1786 (7.2)	356 (6.3)	-	-	-
1992	6031 (8.2)	3434 (8.3)	2149 (8.7)	439 (7.8)	-	-	-
1997	8175 (11.2)	4550 (11.0)	2952 (11.9)	634 (11.2)	-	-	-
2002	8453 (11.5)	4638 (11.2)	2986 (12.1)	754 (13.3)	-	-	-
2007	6175 (8.4)	2612 (6.3)	2797 (11.3)	565 (10.0)	4367 (17.1)	1117 (22.6)	531 (12.1)

2012	5670 (7.7)	2586 (6.3)	2432 (9.8)	537 (9.5)	3530 (13.8)	1049 (21.2)	939 (21.3)
**Insomnia**							
Never	41 314 (57.6)	-	-	-	15 154 (60.0)	1573 (32.4)	2795 (64.8)
Sometimes	24 749 (34.5)	-	-	-	8797 (34.8)	1978 (40.7)	1325 (30.7)
Often	5655 (7.9)	-	-	-	1292 (5.1)	1310 (26.9)	193 (4.5)
**Night sleep duration**							
7-8 h	25 589 (73.3)	15 154 (77.6)	8797 (72.7)	1292 (46.2)	-	-	-
Short, <7 h	4938 (14.1)	1573 (8.1)	1978 (16.3)	1310 (46.9)	-	-	-
Long, >8 h	4405 (12.6)	2795 (14.3)	1325 (11.0)	193 (6.9)	-	-	-
**Insomnia PRS**	0.0 (1.0)	-0.06 (0.99)	0.04 (1.00)	0.15 (1.02)	-0.03 (1.00)	0.10 (1.02)	-0.02 (1.00)
**Sleep duration PRS**	0.0 (1.0	0.04 (1.00)	-0.03 (1.00)	-0.11 (1.02)	0.04 (0.98)	-0.15 (1.01)	0.25 (1.00)
**Short sleep PRS**	0.0 (1.0)	-0.05 (1.00)	0.03 (1.00)	0.13 (1.00)	-0.03 (0.99)	0.15 (1.02)	-0.14 (1.00)
**Long Sleep PRS**	0.0 (1.0)	0.00 (0.99)	-0.01 (1.00)	0.00 (1.05)	0.01 (0.98)	-0.05 (1.00)	0.17 (1.02)
**Covariates for additional models**							
**Total cholesterol, *mmol/l***	5.9 (1.3)	5.9 (1.3)	5.9 (1.3)	6.1 (1.3)	6.1 (1.3)	6.1 (1.4)	6.1 (1.4)
**Body mass index, *kg/m²***	26.4 (4.5)	26.2 (4.3)	26.6 (4.6)	27.2 (4.8)	26.2 (4.3)	27.2 (4.9)	26.4 (4.7)
**Systolic blood pressure, *mmHg***	139.0 (20.8)	138.5 (20.3)	139.0 (21.1)	140.7 (21.7)	140.7 (21.1)	142.2 (22.5)	140.7 (22.7)
**Self-reported diabetes**							
No	67 473 (96.8)	38 646 (97.5)	22 477 (96.5)	5040 (94.1)	22 727 (97.1)	4171 (94.5)	3868 (95.8)
Yes	2198 (3.2)	972 (2.5)	822 (3.5)	317 (5.9)	689 (2.9)	244 (5.5)	170 (4.2)
**Physical activity**							
Low	5912 (8.6)	2631 (6.7)	2174 (9.3)	960 (18.4)	1618 (6.7)	632 (13.8)	543 (13.2)
Intermediate	21 016 (30.6)	11 010 (28.1)	7812 (33.5)	1861 (35.7	6648 (27.4)	1450 (31.6)	1311 (31.2)
High	41 801 (60.8)	25 570 (65.2)	13 341 (57.2)	2386 (45.8)	15 975 (65.9)	2513 (54.7)	2258 (54.9)
**Smoking**							
Never	39 490 (54.6)	22 515 (55.1)	13 291 (54.3)	2819 (50.7)	14 258 (56.5)	2484 (51.2)	2675 (61.7)
Former	13 830 (19.1)	7676 (18.8)	4883 (20.0)	1039 (18.8)	4469 (17.7)	911 (18.8)	693 (16.0)
Current	19004 (26.3)	10 667 (26.1)	6288 (25.7)	1706 (30.7)	6540 (25.9)	1453 (30.0)	968 (22.3)
**Depressive mood**							
Never	31 009 (51.7)	23 553 (65.6)	6574 (34.3)	784 (17.6)	8684 (49.9)	935 (34.5)	1414 (49.2)
Sometimes	25 623 (42.7)	11 766 (32.7)	11 171 (58.2)	2404 (53.9)	7796 (44.8)	1372 (50.6)	1256 (43.7)
Often	3384 (5.6)	608 (1.7)	1439 (7.5)	1274 (28.6)	921 (5.3)	1256 (14.9)	205 (7.1)
**Nervousness**							
Never	20 884 (34.6)	16 145 (44.9)	4090 (21.2)	546 (12.1)	5509 (31.4)	665 (24.4)	933 (32.2)
Sometimes	34 607 (57.3)	18 607 (51.7)	13 151 (68.0)	2477 (55.1)	10 531 (60.0)	1534 (55.9)	1683 (58.1)

Often	4861 (8.1)	1207 (3.4)	2095 (10.8)	1474 (32.8)	1505 (8.6)	547 (19.9)	281 (9.7)
**Sleep medication**							
No	21 364 (77.1)	12 729 (90.2)	7612 (69.9)	814 (33.6)	5933 (77.7)	1363 (66.1)	1026 (73.8)
Yes	6339 (22.9)	1385 (9.8)	3280 (30.1)	1606 (66.4)	1699 (22.3)	699 (33.9)	365 (26.2)

[a] Sample with non-missing information for sex and educational class and no prevalent PD. Mean and SD reported for continues variables. Number of individuals and percentage in the sleep characteristics category reported for categorical variables. PD, Parkinson's disease; PRS, polygenic risk score

Supplementary Table S2. Associations of very long sleep (> 9 h) with Parkinson's disease in the cause-specific hazard model (Poisson) in the basic analysis and in the sensitivity analysis.

	Basic analysis			Sensitivity analysis		
	**All**	**PD**	**Cause-specific hazard model (Poisson)[a]**	**All**	**PD**	**Cause-specific hazard model (Poisson)[a]**
**Trait**	No.	No.	IRR (CI 95%)	No.	No.	IRR (CI 95%)
**Night sleep duration**						
<7 h	4938	165	1.01 (0.85-1.19)	2020	102	1.03 (0.84-1.26)
7-9 h	29078	1167	1 (reference)	16 624	870	1 (reference)
>9 h	916	38	1.44 (1.04-1.99)	418	20	1.17 (0.75-1.83)

[a]Model adjusted for sex, educational class, survey year, follow-up time (10-year intervals), and age at the end of follow-up (5-year intervals). PD, Parkinson's disease; CI, confidence interval; IRR, incidence rate ratio

Supplementary Table S3. Associations of sleep characteristics with Parkinson's disease in the cause-specific hazard model (Poisson) and subdistribution hazard model (Fine–Gray) including category of no response.

			**Parkinson's disease**		**Competing risk of death**	
	**All**	**PD**	**Cause-specific hazard model (Poisson)[a]**	**Subdistribution hazard model (Fine-Gray)[b]**	**Cause-specific hazard model (Poisson)[a]**	**Subdistribution hazard model (Fine-Gray)[b]**
**Trait**	No.	No.	IRR (CI 95%)	HR (CI 95%)	IRR (CI 95%)	HR (CI 95%)
**Insomnia**						
Never	42 080	1448	1 (reference)	1 (reference)	1 (reference)	1 (reference)
Sometimes	25 231	954	1.14 (1.05-1.24)	1.06 (0.98-1.15)	1.12 (1.10-1.14)	1.13 (1.10-1.16)
Often	5790	285	1.52 (1.33-1.73)	1.25 (1.10-1.41)	1.39 (1.35-1.43)	1.39 (1.33-1.45)
No response	1810	59	0.87 (0.67-1.14)	0.72 (0.55-0.93)	1.37 (1.31-1.43)	1.40 (1.31-1.50)
**Night sleep time**						
<6.0 h	5024	171	1.02 (0.86-1.20)	0.87 (0.75-1.03)	1.25 (1.21-1.29)	1.30 (1.24-1.36)
6-9 h	25 988	1063	1 (reference)	1 (reference)	1 (reference)	1 (reference)
>=9.0 h	4495	162	1.01 (0.85-1.19)	0.93 (0.79-1.09)	1.19 (1.15-1.23)	1.18 (1.13-1.24)
No response	500/8918	13	1.07 (0.62-1.85)	0.84 (0.48-1.45)	1.56 (1.41-1.73)	1.58 (1.33-1.87)

[a]Model adjusted for sex, educational class, survey year, follow-up time (10-year intervals), and age at the end of follow-up (5-year intervals). [b]Model adjusted for sex, educational class, and survey year. CI, confidence interval; IRR, incidence rate ratio.

Supplementary Table S4. Associations of insomnia symptoms with Parkinson's disease in baseline age groups in the cause-specific hazard model (Poisson).

	Baseline age < 35 years			Baseline age 35–50 years			Baseline age 50-65 years			Baseline age >= 65 years		
**Trait**	No., all	No., PD	IRR (CI 95%)	No., all	No., PD	IRR (CI 95%)	No., all	No., PD	IRR (CI 95%)	No., all	No., PD	IRR (CI 95%)
**Insomnia**												
Never	12 187	256	1 (reference)	16 046	600	1 (reference)	11 218	482	1 (reference)	1863	78	1 (reference)
Sometimes	4705	91	1.08 (0.85-1.37)	9038	333	1.14 (1.00-1.30)	9149	421	1.14 (1.00-1.30)	1857	88	1.26 (0.92-1.72)
Often	649	17	1.66 (1.01-2.71)	1766	79	1.52 (1.20-1.92)	2765	158	1.55 (1.29-1.86)	475	26	1.44 (0.92-2.27)

Model adjusted for sex, educational class, survey year, follow-up time (10-year intervals), and age at the end of follow-up (5-year intervals). CI, confidence interval; IRR, incidence rate ratio.

Supplementary Table S5. Interaction of sleep characteristics and sex in the cause-specific hazard model (Poisson) for Parkinson's disease.

	Sleep*sex interaction
**Trait**	IRR (CI 95%)
**Insomnia**	
Never	1 (reference)
Sometimes	1.12 (0.95-1.32)
Often	1.08 (0.83-1.40)
**Night sleep time**	
<7 h	1.14 (0.81-1.59)
7-8 h	1 (reference)
>8 h	1.04 (0.74-1.45)

Model adjusted for educational class, survey year, follow-up time (10-year intervals), and age at the end of follow-up (5-year intervals). CI, confidence interval; IRR, incidence rate ratio.
